# Review of three Recent Books on the Boundary of Bioinformatics and Systems Biology

**DOI:** 10.1186/1475-925X-9-33

**Published:** 2010-07-14

**Authors:** Eric Bullinger, Monica Schliemann

**Affiliations:** 1 Department of Electrical Engineering and Computer Science (Montefiore Institute) and GIGA (Interdisciplinary Cluster for Applied Geno-proteomics), Université de Liège, Belgium; 2 Institute for Cell Biology and Immunology and Institute for Systems Theory and Automatic Control, Universität Stuttgart, Germany

## 

• *Bioinformatics for Systems Biology *by S. Krawetz (Ed.), Springer, February 2009 (2^nd ^ed.) ISBN: 978-1-934115-02-2 (BSB)

• *Systems Biology and Bioinformatics: A Computational Approach *by K. Najarian, C. Eichelberger, S. Najarian and S. Gharibzadeh, CRC Press, April 2009, ISBN: 9781420046502 (SBB)

• *Biomolecular Networks: Methods and Applications in Systems Biology *by L. Chen, R.-S. Wang and X.-S. Zhang, Wiley, July 2009, ISBN: 978-0470243732 (BN)

Systems biology is a holistic approach combining experimental data on multiple levels, from the genome and proteome over the interactome to signalling and metabolism in single cells, organs and organisms with the use of computational methods and predictive mathematical models. Bioinformatics is the application of computer science to biological systems, in particular for genomic data. This point of view is shared by the three books, published within a few months last year. All three books target a general audience with basic knowledge of biology, mathematics and computer science and cover quite similarly topics. An obvious difference between these books is their size: there is roughly a two-fold increase from SBB to BN and from the latter to BSB, which distinguished itself from the other two by being a collection of chapters by different authors, with different writing and presentation styles.

All books contain an introduction to biology and to the mathematics needed in the remainder. BSB presents in a complete, very detailed and illustrated manner the cellular organisation over gene expression up to protein synthesis and cell signalling, in over 100 pages. These chapters contain glossaries and further references and are even suited for biologists in need of a recap. The introduction also contains a chapter on the epigenetics of spermiogenesis, whose style and specificity seems somehow misplaced in this part of the book, as it is more a research article than a review or textbook article as the other chapters of Part I. Also the chapter "Genomic Analysis of Transcription" might have been better placed in Part III "Transcriptome Analysis".

BN's biological introduction is significantly shorter: just a few pages, contains a few graphical illustrations and is already very directed towards networks, highlighting their presence from gene regulation to signalling. SBB takes a middle road, the introduction is proportionally longer, however the presentation is fundamentally different, almost as a listicle (heading with one or two paragraphs, or even just lists) without graphical illustrations.

Experimental techniques are discussed differently in the three books. SBB devotes a five page chapter, mostly for genetic and gene expression data. BN also contains a very brief description of DNA and CHiP chip, used for measuring mRNA and transcription factor levels. BSB describes the same techniques, however in much more details as three chapters cover microarrays and one CHiP chips, presenting all steps from experiment design to image analysis in much detail and with several illustrations and thus well suited for experimentalists. Missing in all three books are experimental techniques necessary for quantifying protein concentrations or flux measurements, even though these would be required for quantitative measurements of metabolic or signalling pathways.

BSB contains an introduction to statistical tools, covering a wide range of topics: from probability to stochastic modelling. The writing style and targeted audience of these chapters vary heavily; some are suited for experimentalists with very informative, step-by-step calculations, others are written for theoreticians; some are short while others are quite lengthy. For example, the introductions to probability and to microarray design are well suited for biologists, while stochastic model is targeted for theoreticians. Each reader thus needs to pick a personal selection of chapters.

Sequence alignment and annotation are two major application areas of bioinformatics. BSB gives a very introductory and hands-on description, discussing methods and software often in the style of a cookbook. The book is thus a useful tool for novices with little theoretical knowledge in need of step-by-step help. These topics are also covered in SBB, however much more compact and without concrete examples and illustrations as in BSB. BN replaces this area by network alignment, which is not discussed in the other books and fits well to the main strand of this book.

Not surprisingly, networks form the core of BN and many topics in this area are covered in detail and with illustrations throughout. A first part discusses network reconstruction, ie. how to obtain a mathematical network description from experimental data, for gene regulatory, transcription regulation and protein-protein interaction networks. Then topological properties such as motifs and hubs are introduced before dynamical models of metabolic and signalling networks. These chapters require no specific mathematical prerequisites, though a certain ease with mathematical terminology and equations will not do any harm.

BSB devotes a small, relatively compact part to structural and dynamical reconstruction of networks while SBB leaves these topics out, replacing them by metabolic control analysis and system identification methods. This choice puzzles us as they are not well connected to the remainder of the book. SBB's chapter on Binary&Bayesian Networks is particularly short: only 4 pages, no references; and two paragraphs only for Boolean networks without an example. Strikingly, the chapter finishes saying that Bayesian methods are a promising tool.

From a writing style point of view, the three books differ significantly. SBB often has a very telegraphic style, like a commented list of items. Throughout the book, a few topics are treated in more depths and illustrated with case studies, for example of metabolic control, cell cycle modelling and protein folding. These examples help understanding the theory, though only the metabolic control example contains all the numerical values necessary for repeating the calculations. BSB is probably the most accessible book; however the level of details of some chapters might be dissuasive for more experiences readers. BN addresses a more theoretical audience and focuses on the network aspect, both structurally and dynamically. Its writing style is also the most coherent one of the three books.

BSB furthermore contains a CD-ROM with the figures of the book, some of them in colour. Unfortunately, many figures are in rather low resolution, both in the book and on the CD-ROM. In particular, text within the figures is usually not readable. Each figure with corresponding legend is on a pdf with the page header from the book, but without book title or authors. A better formatting and presentation would have simplified their import into lectures or presentations as suggested in the book's preface.

To summarise, BSB gives a detailed overview of classical bioinformatics, and only a small part on networks and a single chapter on dynamical models. Thus, it does not really cover the area of dynamical modelling, a main component of systems biology. For bioinformatics, it is a very valuable reference book with lists of key papers in addition to the normal citations, and in some chapters with further resources. The biological introduction can be used as a textbook while the other parts are more a reference work targeting in part experimentalists, in part theoreticians.

BN focuses, as the title suggests, on networks from the genome and the proteome level to metabolism and signalling. For each level, the corresponding types of networks and their computational tools and methods are presented in a way that is more suited to a theoretical audience. Nevertheless, the theoretical questions and methods are always put into relation to biology. The book could be used as a textbook, even though there are no problem/exercise sections. It would have profited from "further reading" sections.

SBB is designed as a textbook and is suitable for students of a theoretical background that need an all-round view of systems biology. Its compactness and not too detailed material is probably preferable for undergraduate teaching. It is less recommended for more advanced users, as the coverage is not very deep and the choice of topics seems biased.

The comparison of the three books uncovers the two main strands of systems: On the one hand, the combination of different data across levels or from large scale, e.g. genome-wide experiments; on the other emerging properties of dynamical systems, requiring dynamical mathematical modelling. The first aspect is mostly present in bioinformatics and high-throughput approaches and more or less covered in all three books. The dynamical aspect is more prevalent in BN and SBB. Readers interested in dynamical modelling of biological systems will prefer other books, e.g. [1] while the network motif aspect is the core of [2]. Every one of the three books reviewed here has extra topics that reflect the particular interests of the respective authors and stick out in this comparative study.

## Competing interests

The authors declare that they have no competing interests.

## Authors' contributions

Both authors contributed equally
